# Novel Urethane-Dimethacrylate Monomers and Compositions for Use as Matrices in Dental Restorative Materials

**DOI:** 10.3390/ijms21072644

**Published:** 2020-04-10

**Authors:** Izabela M. Barszczewska-Rybarek, Marta W. Chrószcz, Grzegorz Chladek

**Affiliations:** 1 Department of Physical Chemistry and Technology of Polymers, Silesian University of Technology, 44-100 Gliwice, Poland; Marta.Chroszcz@polsl.pl; 2 Institute of Engineering Materials and Biomaterials, Silesian University of Technology, 44-100 Gliwice, Poland; Grzegorz.Chladek@polsl.pl

**Keywords:** dental restorative materials, urethane-dimethacrylates, degree of conversion, polymerization shrinkage, water sorption, flexural properties, hardness

## Abstract

In this study, novel urethane-dimethacrylate monomers were synthesized from 1,3-bis(1-isocyanato-1-methylethyl)benzene (MEBDI) and oligoethylene glycols monomethacrylates, containing one to three oxyethylene groups. They can potentially be utilized as matrices in dental restorative materials. The obtained monomers were used to prepare four new formulations. Two of them were solely composed of the MEBDI-based monomers. In a second pair, a monomer based on triethylene glycol monomethacrylate, used in 20 wt.%, was replaced with triethylene glycol dimethacrylate (TEGDMA), a reactive diluent typically used in dental materials. For comparison purposes, two formulations, using typical dental dimethacrylates (bisphenol A glycerolate dimethacrylate (Bis-GMA), urethane-dimethacrylate (UDMA) and TEGDMA) were prepared. The monomers and mixtures were tested for the viscosity and density. The homopolymers and copolymers, obtained via photopolymerization, were tested for the degree of conversion, polymerization shrinkage, water sorption and solubility, hardness, flexural strength and modulus. The newly developed formulations achieved promising physico-chemical and mechanical characteristics so as to be suitable for applications as dental composite matrices. A combination of the MEBDI-based urethane-dimethacrylates with TEGDMA resulted in copolymers with a high degree of conversion, low polymerization shrinkage, low water sorption and water solubility, and good mechanical properties. These parameters showed an improvement in relation to currently used dental formulations.

## 1. Introduction

The world population growth, population ageing, high expectations of reconstruction quality and aesthetics, as well as increasing dental health awareness result in a continuous increase in the demand for composite dental restorative materials [1,2]. To address these challenges, dental composites need to undergo a continuous evolution to provide the best possible structural, physico-chemical, mechanical and biological performance.

**Scheme 1:**
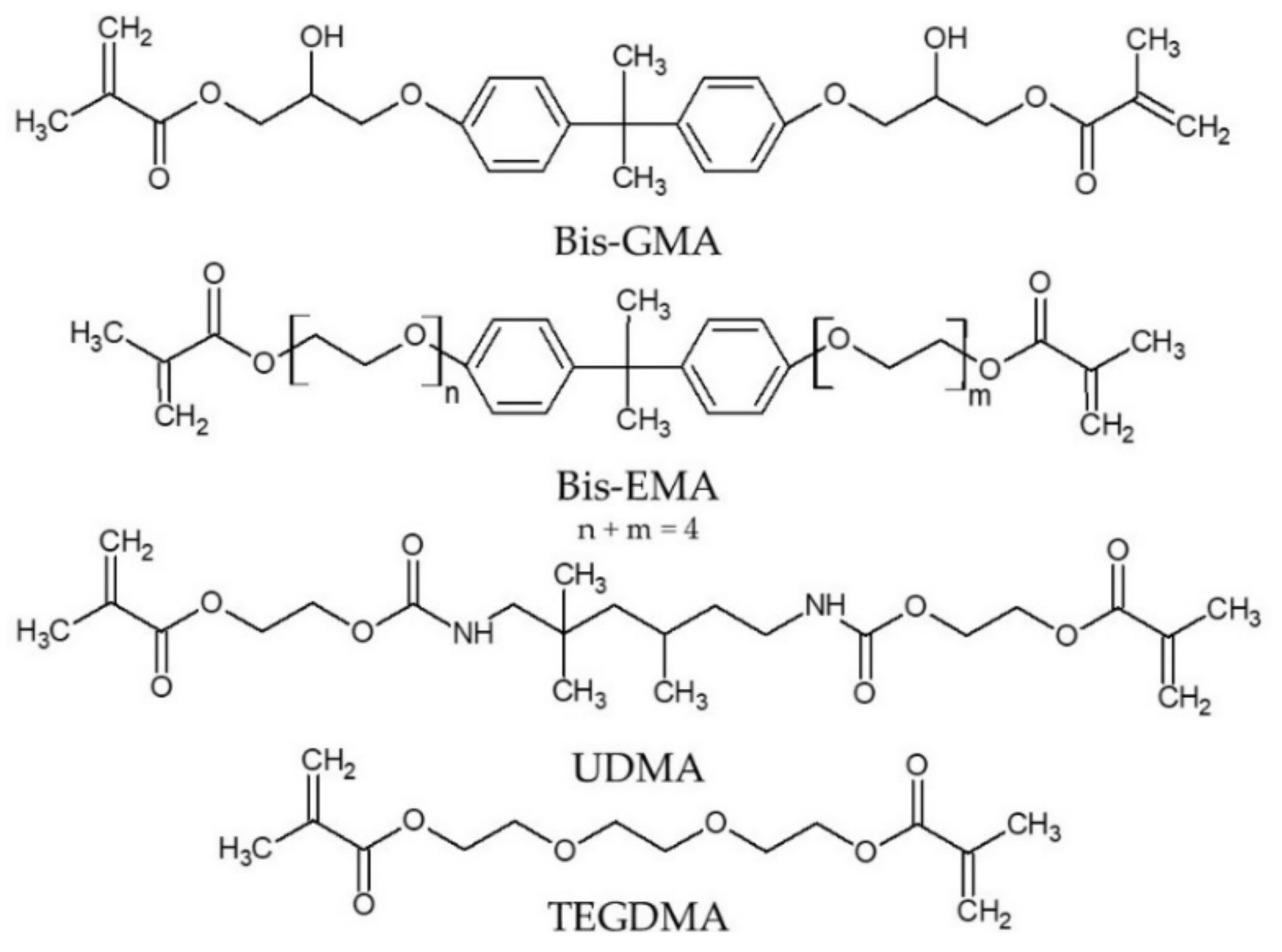
The chemical structure of typical dental dimethacrylates.

The market of dental restorative materials is dominated today by the use of dimethacrylate matrix composites [3,4]. Currently, 86% of them contain at least one of the bisphenol A (BPA)-based monomers [5]. The most significant materials include bisphenol A glycerolate dimethacrylate (Bis-GMA) and bisphenol A ethoxylate dimethacrylate (Bis-EMA) (Scheme 1). The main advantages of Bis-GMA include the relatively low polymerization shrinkage, suitable mechanical properties and excellent adhesion to enamel. The disadvantages of this compound include an extremely high viscosity and low degree of conversion (DC) [6,7,8], which impose the need for mixing the Bis-GMA with other, less viscous monomers, such as reactive diluents. Typically, triethylene glycol dimethacrylate (TEGDMA) is used for this purpose (Scheme 1) [8]. TEGDMA causes a viscosity reduction, which allows for a higher filler loading and excellent cavity filling during application [8]. TEGDMA also positively affects the polymer structure of the composite matrix by increasing the DC and morphological homogeneity, resulting from the microgels’ formation and their agglomeration [9,10,11]. Conversely, TEGDMA is responsible for a decrease in the mechanical strength [12], along with an increase in polymerization shrinkage [4] and water sorption [13,14]. These limitations led to the development of other dimethacrylate resins, such as Bis-EMA. This has a significantly lower viscosity and water sorption than Bis-GMA, polymerizes to a higher *DC*, and has a higher mechanical strength [11]. However, it is more elastic than Bis-GMA [11] and has a worse adhesion to the enamel [4].

The scale of the Bis-GMA and Bis-EMA usage in commercial dental materials is likely to decrease in the coming years, as the products of their biodegradation, such as bisphenol A, bisphenol A diglycidyl ether and bisphenol A dimethacrylate, have estrogenic-like effects [15,16]. Therefore, to reduce human exposure to BPA and its derivatives, research on the development of new BPA-free dental formulations is required.

At present, 1,6-bis-(methacryloyloxy-2-ethoxycarbonylamino)-2,4,4-trimethylhexane, called the urethane-dimethacrylate monomer (UDMA) (Scheme 1), is the only commercial alternative to the BPA-based dental dimethacrylates [11]. UDMA has a significantly lower viscosity than Bis-GMA; however, this is still too high and requires the addition of a reactive diluent [8]. Nevertheless, there are examples of commercial dental composite materials where UDMA is used alone [17]. In comparison to Bis-GMA, UDMA has a lower molecular weight, which results in a slightly higher polymerization shrinkage [8,18]. As it does not contain hydroxyl groups, a reduced water sorption is observed [11,13]. Due to the presence of urethane groups, the UDMA molecule is more flexible than that of Bis-GMA. It also has a higher degree of conversion [19] and morphological homogeneity [9,10,11,12], lower residual monomer content [13] and better mechanical properties [11] of the resulting polymer network. Strong hydrogen bonds, formed by the urethane proton donor group, cause a further increase in the mechanical properties of the composite [11,20,21]. Moreover, UDMA was found to be less toxic than Bis-GMA [21]. A list of the UDMA advantages encourages one to direct research on dental composites towards the development of new urethane-dimethacrylate resins.

There are 24 urethane-dimethacrylate monomers [18,22,23], their corresponding homopolymers [18,24] and copolymers [25] described in the literature. The monomers were obtained using an addition reaction of 2-hydroxyethyl methacrylate (HEMA) and di- (DEGMMA), tri- (TEGMMA) and tetraethylene (TTEGMMA) glycol monomethacrylates with six commercial diisocyanates: 1,6-diisocyanatohexane (HMDI), 2,2,4-trimethyl-1,6-diisocyanatohexane (TMDI), isophorne diisocyanate (IPDI), 4,4’-diisocyanatodicyclohexylmethane (CHMDI), 2,4-diisocyanatotoluene (TDI) and bis-(4-isocyanatophenyl)methane (MDI). The addition reaction of HEMA to TMDI results in the abovementioned UDMA monomer. Despite numerous advantages, the remaining monomers from the series contain certain flaws. The solid-state nature of the monomers with the lowest molecular weights is the most problematic. On the other hand, monomers synthesized from TEGMMA and TTEGMMA are usually too elastic, which results in an insufficient modulus and excessive water sorption. The most promising physico-mechanical properties emerged with the following urethane-dimethacrylates: HEMA/IPDI, DEGMMA/IPDI, DEGMMA/CHMDI and TEGMMA/TDI [18].

Moszner et al. used different commercial diisocyanate – 1,3-bis(1-isocyanato-1-methylethyl)benzene (MEBDI) to develop a series of new urethane-dimethacrylates [26,27]. These were synthesized by an addition reaction of MEBDI to HEMA, 2-hydroxypropyl methacrylate, 2-hydroxy-3-phenoxypropyl methacrylate and glycerol dimethacrylate. The products of their polymerizations were tested for their flexural properties and water sorption. The results showed that the addition product of HEMA to MEBDI had satisfactory properties and could potentially replace Bis-GMA in dental composites [26,27].

**Scheme 2:**
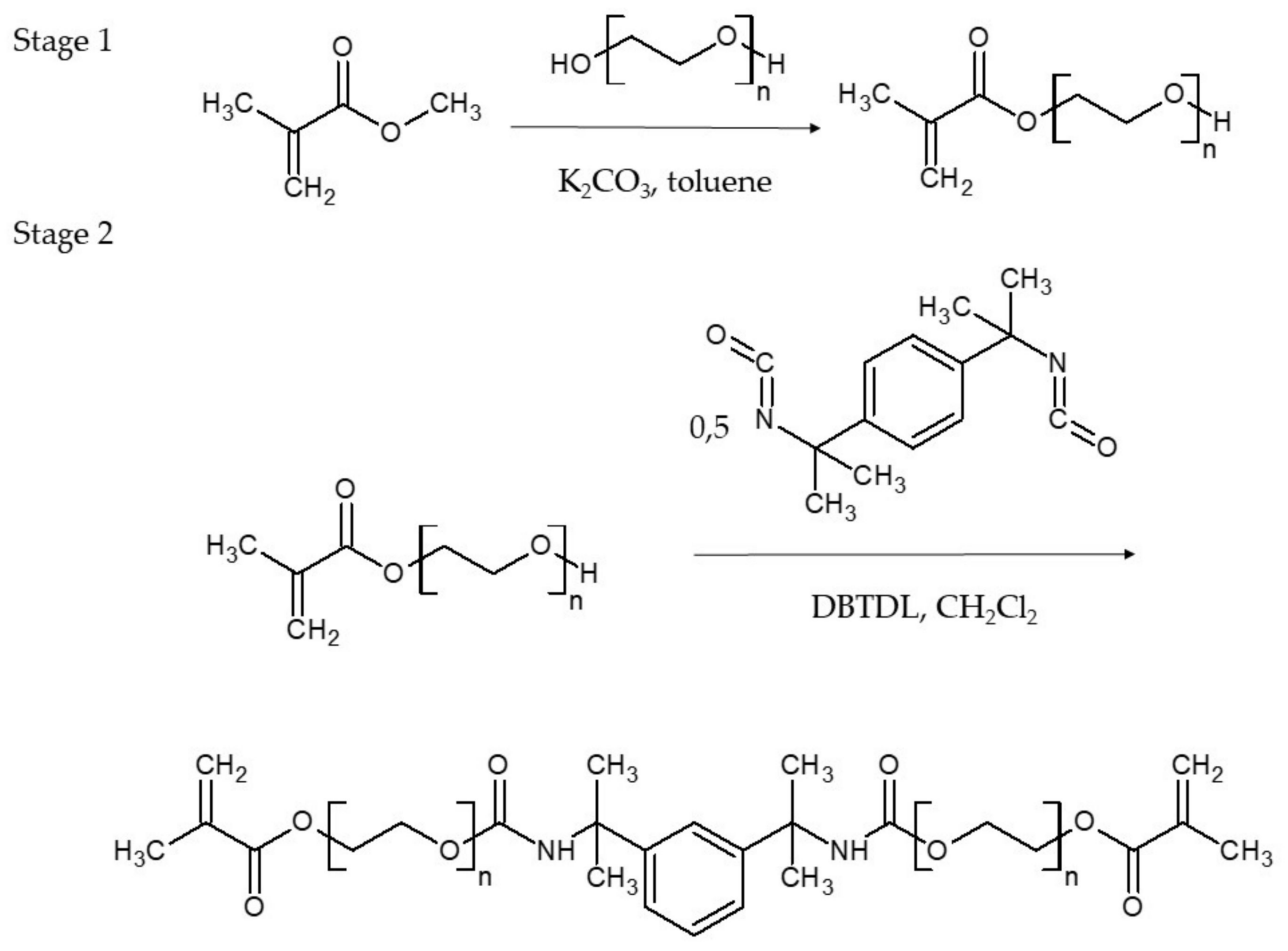
The synthesis route and chemical structure of the MEBDI-based urethane-dimethacrylates synthesized in this study (HM – *n* = 1, DM – *n* = 2 and TM – *n* = 3).

This work aimed at the development of new BPA-free resin formulations, based on new urethane-dimethacrylates, that could offer similar or better physico-mechanical properties than those which are based on Bis-GMA, UDMA and TEGDMA. The overall very good properties of the dimethacrylates and their low manufacturing costs still inspire scientists to design new monomers of this type [18,22,23,26,27,28,29,30,31]. However, the narrow list of commercial dimethacrylates suggests that there is still no strong alternative to them. Moreover, the proportion of alternative dimethacrylates is expected to increase in parallel with a reduction in the use of the BPA-based resins. Therefore, the development of new BPA-free monomers, which would provide adequate physico-mechanical characteristics of the composite in combination with low manufacturing costs, are of huge interest. In this study, the addition reactions of MEBDI to HEMA, DEGMMA and TEGDMA were used to synthesize a series of urethane-dimethacrylates (Scheme 2). These monomers were then mixed in various ratios, which were specified in Table 1. The monomers and mixtures were tested for their viscosity and density. The polymers were characterized by their degree of conversion, polymerization shrinkage, water sorption and solubility, flexural properties and hardness.

## 2. Results

### 2.1. Monomer Characterization

Monomers and monomer mixtures were tested for viscosity and density. These results, as well as the monomer molecular weights and concentrations of double bonds, are summarized in Table 2.

As can be seen from Table 2, the MEBDI-based monomers showed density values (d_m_) from 1.12 to 1.15 g/cm^3^. This means that these monomers have a similar density to Bis-GMA and are denser than UDMA and TEGDMA. The MEBDI-based compositions’ viscosity values ranged from 1.13 to 1.14 g/cm^3^, which directly overlaps with the range from the dental dimethacrylate compositions.

HM, DM and TM were resinous in character. However, the viscosity (η) of HM was too high, and it was not possible to measure it at room temperature. The viscosity determination for the entire series of monomers was carried out at two temperatures, 20 °C and 45 °C. The determined values ranged, respectively, from 2.58 to 19.84 Pa∙s (excluding HM) and from 0.52 to 18.75 Pa∙s. It was found that the longer the oligooxyethylene chain, the lower the viscosity. A comparison of these *η* values to those of dental dimethacrylates resulted in the following order of increasing viscosity: T < TM < U < DM < HM < Bis-G. These increases were all statistically significant (*p* ≤ 0.05). The viscosity data for Bis-GMA and TEGDMA were taken from the literature [19]. As can be seen, the viscosities of the MEBDI-based monomers are between those of TEGDMA and Bis-GMA. When compared to UDMA, HM and DM were more viscous, whereas TM was less viscous.

The mixtures of the MEBDI-based monomers exhibited viscosity values ranging from 2.82 to 7.25 Pa∙s (at 20 °C) and from 0.85 to 1.46 Pa∙s (at 45 °C). From a comparison of these values measured at 20 °C to those obtained for dental dimethacrylate compositions, the following order could be constructed: Bis-G:T < HM:DM:T < HM:T < HM:DM:TM < Bis-G:U:T < HM:TM. These differences showed a statistical significance (*p* ≤ 0.05). The analogous comparison of *η* values determined at 45 °C led to the construction of the following order: Bis-G:T < HM:DM:T < HM:DM:TM < HM:T < HM:TM < Bis-G:U:T. These results were usually statistically significant (*p* ≤ 0.05), except the results for the HM:T and HM:DM:TM, as well as the results for the HM:DM:T and HM:DM:TM couples, which were statistically insignificant (*p* > 0.05). In summary, the viscosities of the MEBDI-based formulations ranged between the viscosities of Bis-G:T and Bis-G:U:T, except for the viscosity of HM:TM. Its value, determined at room temperature, was 23% higher than that of Bis-G:U:T.

### 2.2. Degree of Conversion

**Figure 1:**
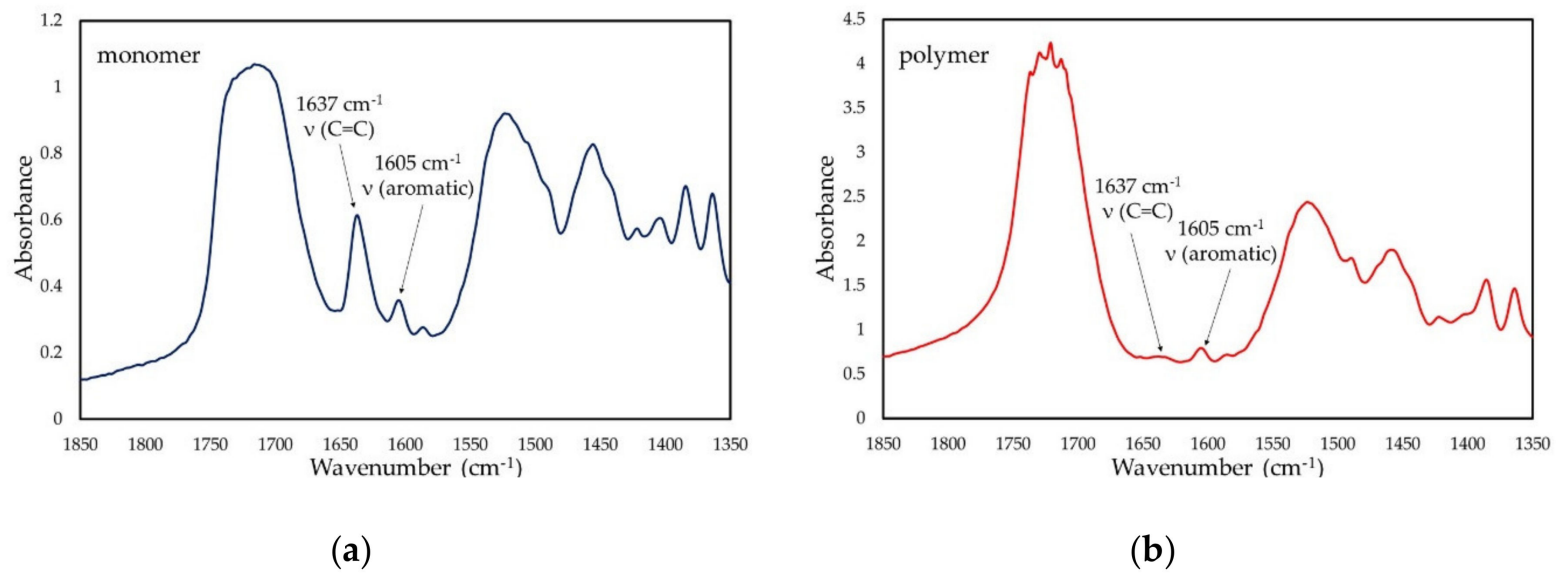
The representative FTIR spectra of the DM: (**a**) monomer and (**b**) polymer.

The DC was determined utilizing the FTIR internal standard method [11,32]. The absorbance of the band corresponding to the C=C vinyl stretching vibrations was ratioed to the absorbance of the band corresponding to the aromatic stretching vibrations, in the resins and their cured forms. Representative FTIR spectra of the DM monomer is shown in Figure 1a and polymer - in Figure 1b. The average DC values of all the studied samples are summarized in Table 3.

As can be seen from Table 3, the DC in the MEBDI-based homopolymers ranged from 57.7 to 89.0%. HM achieved the lowest DC. The DC values determined for DM and TM (89.0% and 88.4%, respectively) were statistically significantly higher than that of HM (*p* ≤ 0.05). A statistical significance was not observed between the results for DM and TM (*p* > 0.05). When comparing the DC values of the MEBDI-based homopolymers and dental dimethacrylates, the following increasing order could be constructed: Bis-GMA < HM < UDMA < TEGDMA < TM < DM. The DC data for Bis-GMA and TEGDMA were taken from the literature [33]. The *DC* value that emerged from HM was statistically significantly lower than that of UDMA (*p* ≤ 0.05), but it was higher than that of Bis-GMA. It is worth noting that homopolymerizations of DM and TM led to DC values higher than those of Bis-GMA, UDMA and TEGDMA.

The DC in the MEBDI-based copolymers ranged from 65.2% to 80.7%. A comparison of these DC values to those of the dental dimethacrylate copolymers resulted in the following increasing order of DC: Bis-G:T < HM:TM < Bis-G:U:T < HM:DM:T < HM:T < HM:DM:TM. The DC values of the MEBDI-based copolymers were usually higher than those of the dental dimethacrylate copolymers, except for HM:TM. This DC value was lower than that of Bis-G:U:T and higher than that of Bis-G:T. However, these differences were statistically insignificant (*p* > 0.05). The HM:DM:TM showed the highest DC, and its value was statistically significant (*p* ≤ 0.05) in comparison to the remaining copolymers. From these observations it can be summarized that new MEBDI-based formulations occur with an efficiency similar to that of dental dimethacrylate formulations.

### 2.3. Polymerization Shrinkage

Table 3 shows the results for the experimentally determined polymerization shrinkage (S) and theoretically calculated polymerization shrinkage (S_theor_). The latter was calculated from the monomer density (d_m_), assuming a DC of 100% and a volumetric shrinkage of 22.5 cm^3^ for the polymerization of one mole of double bonds [34].

The MEBDI-based urethane-dimethacrylates showed an *S* from 3.03% to 4.31% and S_theor_ from 7.83% to 10.25%. HM had the lowest *S* and the highest S_theor_. S initially increased, as the number of oxyethylene groups increased, and finally slightly decreased. These results were statistically significant (*p* ≤ 0.05). S_theor_ decreased as the length of the oligooxyethylene chain increased within the whole series. When comparing the S values of MEBDI-based homopolymers with those of dental dimethacrylates, the following increasing order could be constructed: U < HM < TM < DM < Bis-G < T. As can be seen, the S values from MEBDI-based monomers were between those of UDMA and Bis-GMA (and TEGDMA). The differences between UDMA and HM were statistically insignificant (*p* > 0.05); however, all the rest were statistically significant (*p* ≤ 0.05). A comparison of the MEBDI-based homopolymers and dental dimethacrylates with respect to their S_theor_ resulted in the following order: TM < DM < Bis-G < HM < U < T. It can be seen that TM and DM are characterized by the lowest theoretical volumetric contraction. HM has a S_theor_ value lower than those of UDMA and TEGDMA, but higher than that of Bis-GMA. The general assessment of the results for *S* and S_theor_ suggests that the higher the *S,* the lower the S_theor_; this is not observed for TEGDMA, which is the smallest molecule and polymerizes with a very high *DC*.

When comparing the copolymers for their value of S, the following order can be determined: HM:DM:T < HM:T < HM:TM < Bis-G:U:T < Bis-G:T < HM:DM:TM. It can be seen that HM:DM:T was characterized by the statistically lowest S (*p* ≤ 0.05). The S values of HM:T and HM:TM were statistically similar to those of Bis-G:U:T (*p* > 0.05). HM:DM:TM showed the highest *S* value, which was statistically similar to that of Bis-G:T (*p* > 0.05). In summary, the MEBDI-based formulations usually had a lower *S* than the dental formulations. If the mixtures are compared using their theoretical values of polymerization shrinkage, they can be ordered accordingly: HM:DM:TM < HM:TM < HM:DM:T < HM:T < Bis-G:U:T < Bis-G:T. This shows that both dental formulations have a higher S_theor_ than for each of the MEBDI-based mixtures.

### 2.4. Water Sorption and Solubility

In Table 4, the results for water sorption (WS) and solubility (SL) are summarized. The upper thresholds of these parameters, for the dental polymer-based restorative materials, are specified according to the ISO 4049 standard [35]. From ISO 4049, a composite dental material must have a water sorption lower than 40 µg/mm^3^ and a solubility lower than 7.5 μg/mm^3^.

The WS values of the MEBDI-based homopolymers ranged from 13.34 to 46.01 µg/mm^3^. It was observed that the longer the oligooxyethylene chain, the higher the WS. These increases in *WS* values showed a statistical significance (*p* ≤ 0.05). A comparison of the copolymers resulted in the following trend: U < HM < DM < Bis-G < TM < T. The *WS* of the new MEBDI-based monomers were between the *WS* of UDMA and TEGDMA. HM exhibited the lowest WS amongst the MEBDI-based homopolymers; however, its value did not show a statistical significance with respect to UDMA (*p* > 0.05). The remaining results obtained for the *WS* of the homopolymers were all statistically significant (*p* ≤ 0.05). The *WS* data for Bis-GMA and TEGDMA were taken from the literature [13]; therefore, their statistical significance could not be assessed. HM and DM were at lower *WS* values than that of Bis-GMA, whereas TM had a higher *WS* value with respect to Bis-GMA. The *WS* value for TM was 46 µg/mm^3^, which is 15% higher than the limiting value of 40 µg/mm^3^ specified in ISO 4049. It should be noted that TEGDMA showed a *WS* value of 66.93 µg/mm^3^, which corresponds to a 67% excess over the limit specified in ISO 4049.

A comparison of the MEBDI-based monomers and dental dimethacrylates by WS yielded the following order: HM:T < HM:DM:T < Bis-G:U:T < Bis-G:T < HM:DM:TM < HM:TM. Both MEBDI-based copolymers containing TEGDMA had the lowest WS values. The difference in the WS values of HM:T and HM:DM:T did not show a statistical significance (*p* > 0.05). However, these results showed a statistical significance with respect to both dental copolymers and both MEBDI-based copolymers containing TM (*p* ≤ 0.05). The latter couple showed WS values higher than both the dental copolymers. However, the results for Bis-G:T, Bis-G:U:T and HM:DM:TM were not statistically significant (*p* > 0.05). HM:TM showed the highest WS value at 32.67 µg/mm^3^. This result was statistically significant with respect to the results for the remaining copolymers (*p* ≤ 0.05). In summary, the MEBDI-based compositions usually had a similar or lower *WS* when compared to dental resin compositions, the exception being HM:TM. However, it is noteworthy that none of the MEBDI-based compositions had a WS value higher than 40 µg/mm^3^, which is specified by the ISO 4049 standard as the upper threshold.

As can be seen from Table 4, the SL values of the MEBDI-based homopolymers ranged from 3.13 to 6.31 µg/mm^3^. The SL initially decreased, as the number of the oxyethylene units increased from one to two, and then they began to increase, as the number of the oxyethylene units increased up to three. When considering dental dimethacrylates, the following increasing order of SL was observed: T < HM < U < TM < DM < Bis-G. The solubility of the MEBDI-based monomers was between the solubilities of TEGDMA and Bis-GMA. HM had a lower solubility than UDMA, whereas DM and TM had a greater solubility than UDMA. All the results obtained from the homopolymers were statistically significant (*p* ≤ 0.05). The presented data for Bis-GMA and TEGDMA were taken from the literature [13]; therefore, their statistical significance could not be assessed.

A comparison of the SL values for the copolymers of the MEBDI-based monomers and dental dimethacrylates resulted in the following order: HM:DM:TM < Bis-G:T < HM:TM < Bis-G:U:T < HM:T = HM:DM:T. The MEBDI-based copolymers containing TM showed a lower water solubility than those containing TEGDMA. HM:DM:TM had the lowest water solubility and showed an SL value significantly lower than those of both dental copolymers (*p* ≤ 0.05). The *SL* value of HM:TM was lower than that of Bis-G:U:T, but higher than that of Bis-G:T. These results were also shown to be statistically significant (*p* ≤ 0.05). HM:T and HM:DM:T showed the same SL values (*p* > 0.05). These values were statistically significantly higher than those of both dental copolymers (*p* ≤ 0.05). None of the studied copolymers showed an *SL* value higher than 7.5 µg/mm^3^, which is specified by the ISO 4049 standard as the upper threshold.

### 2.5. Mechanical Properties

In Table 5, the results for flexural modulus (E), flexural strength (σ) and hardness (HB) are summarized.

The modulus of the MEBDI-based homopolymers ranged from 3885 to 4657 MPa, whereas the flexural strength ranged from 87.4 to 164.4 MPa. It can be observed that *E* and *σ* depend on the length of the oligooxyethylene chain. The higher the number of oxyethylene units, the lower the *E* and the higher the *σ*. These differences showed statistical significance (*p* ≤ 0.05).

When comparing the homopolymers of the MEBDI-based monomers and dental dimethacrylates by their *E* value, the following increasing order could be constructed: U < Bis-G < TM < T < DM < HM. MEBDI-based homopolymers usually had a higher stiffness than the dental dimethacrylates, except in the case of TM. The E value for TM was higher than those of Bis-GMA and UDMA, but lower than that of TEGDMA. When the achieved *σ* values by the MEBDI-based homopolymers were compared to those of dental dimethacrylates, the following increasing order could be constructed: U < HM < T < Bis-G < DM < TM. The MEBDI-based homopolymers usually had higher *σ* values than the dental dimethacrylates, except for the HM. The σ value for HM was higher than that of UDMA, but lower than those of Bis-GMA and TEGDMA. All results obtained for the flexural properties of the homopolymers showed a statistical significance. The significance of the results for Bis-GMA and TEGDMA could not be assessed as their *E* and *σ* values were taken from the literature [18].

A comparison of the copolymers by their *E* values resulted in the following trend: Bis-G:T < Bis-G:U:T < HM:T < HM:TM < HM:DM:T < HM:DM:TM. As can be seen, both dental copolymers showed lower E values than all the MEBDI-based copolymers. These results were statistically significant (*p* ≤ 0.05). The analysis of differences in E within the group of the MEBDI-based copolymers usually revealed statistical insignificance (*p* > 0.05), except for HM:DM:TM. This showed a statistically significantly higher E value than for the HM:T and HM:TM (*p* ≤ 0.05).

A comparison of the copolymers by *σ* resulted in the following increasing trend: Bis-G:T < HM:T < Bis-G:U:T < HM:TM < HM:DM:T < HM:DM:TM. It can be seen that the MEBDI-based copolymers usually exhibited higher *σ* values when compared to the copolymers of dental dimethacrylates, except for HM:T. These differences were statistically significant (*p* ≤ 0.05). The *σ* value of HM:T was statistically insignificantly lower than that of Bis-G:U:T (*p* > 0.05) and was statistically significantly higher than that of Bis-G:T (*p* ≤ 0.05). The differences in *σ* within the group of the MEBDI-based copolymers were statistically insignificant (*p* > 0.05), except for HM:T. This copolymer was characterized by a statistically significantly lower *σ* value than the remaining MEBDI-based copolymers (*p* ≤ 0.05).

The hardness of the MEBDI-based homopolymers ranged from 151.5 to 179.4 MPa. As shown in Table 5, the initial lengthening of the oligooxyethylene chain caused an increase in the HB. The incorporation of a third oxyethylene group caused a decrease in the HB. These differences showed a statistical significance (*p* ≤ 0.05). A comparison of the HB for homopolymers of MEBDI-based monomers and dental dimethacrylates resulted in the following order: Bis-G < T < HM < TM < U < DM. It can be seen that the DM homopolymer was the hardest. However, the differences in HB values for DM, UDMA and TM did not show a statistical significance (*p* > 0.05). HM exhibited a statistically significantly lower HB value than that of UDMA (*p* ≤ 0.05). The significance of the results for Bis-GMA and TEGDMA could not be assessed, as their *HB* values were taken from the literature [18].

A comparison of the HB for the copolymers resulted in the following trend: Bis-G:T < HM:T < HM:DM:T < HM:TM < Bis-G:U:T < HM:DM:TM. As can be seen, the HB values of the MEBDI-based copolymers typically appeared between those of Bis-G:T and Bis-G:U:T, except for HM:DM:TM. The HM:DM:TM had the statistically significantly highest *HB* value (*p* ≤ 0.05). HM:T had the lowest HB value amongst the MEBDI-based copolymers, which was lower than that of Bis-G:U:T and higher than that of Bis-G:T. All results for HM:T were statistically significant (*p* ≤ 0.05). The results for Bis-G:U:T, HM:TM and HM:DM:T were statistically insignificant (*p* > 0.05).

## 3. Discussion

The purpose of this work was to develop new, BPA-free, urethane-dimethacrylate monomers and their formulations, with a TEGDMA reduction or elimination, achieving high curing efficiency, low polymerization shrinkage and water sorption, whilst giving a good mechanical performance. The MEBDI-based urethane-dimethacrylates are promising materials for achieving these aims.

In this study, we synthesized three UDMA analogues: HM, DM and TM (Scheme 2), to produce new urethane-dimethacrylate monomers and their formulations with properties similar or better to those that are currently in utilized dental formulations. UDMA is commonly used as a dentistry monomer and derives from the urethane-dimethacrylates family. It is obtained by an addition reaction between TMDI and HEMA. In this work, a series of novel urethane-dimethacrylates were synthesized by replacing TMDI with MEBDI. Three oligooxyethylene monomethacrylates of various lengths (HEMA, DEGMMA and TEGMMA) were used as the second substrate. It must be noted that the monomer obtained from HEMA and MEBDI was known from the literature, as it was previously described by Moszner et al. [25,26].

The obtained MEBDI-based monomers were compounded with themselves and TEGDMA to prepare four new formulations (Table 1). Two of them were solely composed of the MEBDI-based monomers (HM:TM and HM:DM:TM). In a second pair (HM:T and HM:DM:T), TM was replaced with TEGDMA, a reactive diluent typically used in dental materials. TEGDMA and TM were in a 20 wt.%. For two further samples, HM was diluted with DM at a 1:1 weight ratio. For comparison purposes, two typical dental formulations were prepared. They consisted of Bis-GMA and TEGDMA, mixed in the 60:40 weight ratio (Bis-G:T), and Bis-GMA, UDMA and TEGDMA, mixed in the 40:40:20 weight ratio (Bis-G:U:T).

Urethane-dimethacrylates obtained from HEMA and aromatic diisocyanates are typically crystalline solids [18]. The planar geometry of the benzene ring is responsible for the tight packing of the monomer molecules, thus limiting their mobility and facilitating a crystalline network formation [24]. The resinous character of the MEBDI-based monomers can be attributed to the presence of the spacious MEBDI substituents. However, the presence of aromatic rings and urethane bonds resulted in a very high viscosity of the HM (18.75 Pa∙s, measured at 45 °C). This was so high that it could not be measured at room temperature. As the length of the oligooxyethylene chain increased, the molecular elasticity and mobility increased, and a decrease in the *η* was observed. The viscosity value determined at 20 °C for DM (19.84 Pa∙s) was 2.35 times higher than that of UDMA (8.44 Pa∙s), whereas the viscosity of TM (2.58 Pa∙s) was 3.27 times lower than that of UDMA. None of the MEBDI-based dimethacrylates were as viscous as Bis-GMA, which can be attributed to the lack of hydroxyl groups in their structures.

According to previous literature, it can be assumed that the UDMA viscosity is the highest that is possible in order for a monomer to be used alone in a dental formulation [17]. Therefore, the application of HM and DM as a single resin dental composite matrix cannot be recommended. TM exhibited a viscosity sufficiently low enough to be used as a stand-alone resin in a dental composite matrix. As TM was significantly more viscous than TEGDMA (0.01 Pa∙s [19]), it could not replace TEGDMA as a reactive diluent.

As HM and DM showed a higher viscosity than UDMA, in order for them to be used in dental composite matrices they have to be compounded with fewer viscous monomers. As expected, the compounding of HM with DM, TM and TEGDMA resulted in a viscosity decrease. For example, the viscosity values of HM:T and HM:TM determined at 45 °C were on average 93% lower than those of HM. When comparing the viscosity values determined at 20 °C for HM:TM and HM:DM:TM with those of HM:T and HM:DM:T, it can be seen that the latter compositions showed 44% lower *η* values than their TM-based counterparts. This result was expected as TM has a viscosity that is three orders of magnitude greater than that of TEGDMA. By comparing the results of the viscosity between mixtures of the MEBDI-based monomers and dental dimethacrylates, it can be seen that new formulations have a sufficiently low viscosity to be used as dental restorative composites. HM:DM:T appeared to be the most promising, as it had a viscosity 52% lower than that of Bis-G:U:T.

The degree of conversion in the dental composite dimethacrylate matrix is an important parameter, as it determines the physico-chemical and mechanical behavior of the composite [11]. The higher the DC, the higher the polymerization shrinkage [36], the better the mechanical properties [11,37], and the lower the water sorption [13] and monomer leaching [11,37,38]. Theoretically, the minimum DC in a dental composite dimethacrylate matrix should be higher than 50% [8]. The literature also suggests a *DC* lower limit of 55%, which should guarantee the clinical success of a tooth restoration [39].

All homo- and copolymerizations performed in this study resulted in a DC higher than 55%. The same two criteria for curing efficiency were fulfilled.

The lowest DC of the homopolymer HM (57.7%) can be explained by the short length and high rigidity of the monomer molecule, which strongly limits molecular motion and the possibility of reorganization [11]. By lengthening the oligooxyethylene chains, the monomer molecules gain mobility, which resulted in a DC increase. Interestingly, an increase in the number of oxyethylene groups from one to two resulted in a radical increase in the DC. The homopolymer of DM had a DC of 89.0%. The incorporation of a third oxyethylene group into the oligooxyethylene chain caused an insignificant decrease in the DC. This phenomenon is known in the literature and is explained by an increasing distance between the methacrylate groups [32].

For the copolymers, it can be concluded that the compounding of the MEBDI-based monomers usually resulted in higher DC values when compared to those determined for dental resin compositions. HM:T, HM:DM:T and HM:DM:TM all showed similar DCs with an average value of 73%. This means that the overall molecular mobility and steric hindrance in these systems are similar. The results of the statistical analysis allow one to assume that this level of curing can be recognized as being similar to that achieved by Bis-G:U:T. The DC value of HM:TM was lower than that of Bis-G:U:T and, from a statistical point of view, similar to that of Bis-G:T. A comparison of the results for HM:T and HM:TM suggests that the compounding of HM with TEGDMA can have an improved effect on the *DC* when compared to the application of TM. This result can be explained by the greater dimensions of the TM molecule. Smaller TEGDMA molecules have a greater ability to fill free space and, therefore, to form a polymer network of greater homogeneity [9,10,11]. In conclusion, HM:T, HM:DM:T and HM:DM:TM all have a better suitability for potential use in dental applications than HM:TM does, when assessed by their DC.

The degree of conversion is closely related to the experimentally determined polymerization shrinkage (S). The higher the DC, the higher S [40]. The real polymerization shrinkage also depends on the theoretical polymerization shrinkage (S_theor_). In general, the S_theor_ depends on the concentration of double bonds (X_DB_), and their values are summarized in Table 2. As each dimethacrylate molecule has two double bonds, the higher the molecular weight (the lower the concentration of double bonds), the lower the value of S_theor_. In many studies, it has been shown that the polymerization shrinkage can negatively affect the physico-mechanical performance and longevity of dental restoration [41]. For this reason, work on the development of a new dental monomer focuses on the reduced volumetric contraction (a decrease in the volume caused by the formation of a polymer network due to the methacrylate group polymerization).

Similar *S* values were observed for HM and UDMA, which can be attributed to a compensating effect of pairing a low X_DB_ with a high DC. HM has a lower S_theor_ (lower X_DB_) but showed a higher DC than UDMA. Even though DM and TM are characterized by a lower S_theor_, they both resulted in higher S values than that of HM due to the high DC in these homopolymers.

The compounding of the MEBDI-based monomers led to satisfactory results for *S*. The homopolymers usually showed lower *S* values when compared to the dental copolymers. This is due to the lower S_theor_ values of HM:T, HM:TM and HM:DM:T in comparison to Bis-G:T and Bis-G:U:T. The only composition that exhibited a higher volumetric contraction than the dental formulations was HM:DM:TM. Even though this had the lowest S_theor_, it also had the highest DC, which was responsible for the highest experimentally determined volumetric contraction.

Water sorption (WS) is a physical parameter related to the dimensional stability of the dental composites. Dental composites absorb water molecules from oral fluids, giving a volumetric expansion. An excessive expansion can cause damage to the tooth, such as its fracture [42]. Some researchers have shown that a certain water uptake can be beneficial by compensating for the volumetric contraction resulting from polymerization [11]. For this, the volume increase and the water sorption should be balanced by the volume reduction caused by polymerization shrinkage [43]. Therefore, a value of 40 μg/mm^3^ was established as the maximum WS value for the resin-based dental materials, as given in the ISO standard 4049 [35].

The water sorption of dimethacrylate polymer networks is dependent on numerous structural factors. The most important ones include the monomer molecule length and elasticity, the presence of groups willing to form strong hydrogen bonds, the strength of these bonds and the DC of the polymer [11]. An increasing *WS* of the MEBDI-based homopolymers can be explained by an increasing distance between the double bonds and the incorporation of oxyethylene units, causing an increase in the molecular elasticity, and hence an increase in the molecular mobility. By comparing the UDMA and HM, both with the shortest molecules, the DC revealed a more pronounced effect on the WS. A lower *WS* of UDMA can be explained by a higher DC (77.6%). Even though the HM molecule has a lower concentration of double bonds (3.96 mol/kg), the relatively low DC (57.7%) resulted in an 18% higher WS of its homopolymer in comparison to the fully aliphatic and more elastic UDMA homopolymer. The influence of hydrogen bonding on the WS can be seen when homopolymers of TEGDMA and TM are compared. TEGDMA has a higher concentration of double bonds (6.99 mol/kg) than TM (2.94 mol/kg). The DC in both homopolymers was similar and high (higher than 82%). This means that the crosslink density in the TEGDMA polymer network is higher. Theoretically, tighter packing of TEGDMA macrochains should hinder the water swelling. This suggests that the presence of hydrogen bonds formed by urethane groups causes the greater tightening of the TM polymer network that stops a greater number of water molecules from entering into it. As the *WS* of TM was higher than 40 µg/mm^3^, this monomer cannot be used on its own in a potential dental composite. However, it can be mixed with other dimethacrylates, as is done with TEGDMA.

By comparing the results for the MEBDI-based copolymers, it can be observed that TEGDMA had a better impact on WS than TM. Both compositions containing TEGDMA showed lower WS values than both the dental compositions and the MEBDI-based compositions containing TM. This can be explained by the small dimensions of the TEGDMA molecule. TEGDMA used in a 20 wt.% content probably improved the polymer network homogeneity, whereas its overall elasticity and hydrophilicity were governed by the HM and DM chemical structures. The results obtained for the MEBDI-based formulations from the perspective of limiting the WS value, specified in the ISO standard, suggest that each of them could be used as a matrix in dental composites. However, those containing TEGDMA are more suitable.

Water solubility is particularly important for the biological properties and biocompatibility of dental materials. In contrast to a cured composite matrix, which is harmless to the human body, a soluble fraction can cause tissue irritation and have a more serious effect on the organism [44,45]. Therefore, the quantity of soluble fraction (SL) in the dental restorative material should be controlled. According to the ISO 4049 standard, a dimethacrylate composite, in order to be suitable for use as a dental material, must show a water solubility lower than 7.5 μg/mm^3^. As each of the studied MEBDI-based polymers have SL values below this limit, it is assumed that all of them fulfilled that criterion.

Analyzing the results for the homopolymers, it can be seen that the dimensions and stiffness of the monomer molecule are the main factors determining SL. The DC has less impact on this phenomenon. HM was characterized by the lowest SL. This seems surprising as HM had the lowest DC amongst the homopolymers of urethane-dimethacrylates (including the fully aliphatic UDMA). An explanation for this can be found in the high molecular stiffness of the HM polymer network. The limitations in molecular motion, caused by the presence of the benzene ring, prevent the sol fraction from leaching. Conversely, DM showed the highest *SL*, even though it showed the highest DC. This can be attributed to the increased elasticity resulting from the lengthening of the oligooxyethylene chain. The sol fraction can then more easily migrate through the polymer network. A further increase in the oligooxyethylene length resulted in a slight decrease in the SL. TM showed a 7% lower SL value than DM. This can be explained by the trapping of a greater amount of the sol fraction inside the network due to the increased dimensions of the TM monomer molecule.

A comparison of the MEBDI-based copolymers showed that those containing small TEGDMA molecules had a higher *SL* than those containing TM molecules, as these were the longest in the studied systems. The MEBDI-based copolymers containing TEGDMA also had higher SL values than the dental copolymers, which can be explained by a greater stiffness of the Bis-GMA-based copolymer networks as well as trapping of the unreacted monomer molecules by hydrogen bonding with the aid of the Bis-GMA hydroxyl groups. HM:DM:TM had the lowest SL value due to the synergistic effect of the highest DC and the TM presence (the longest monomer used in the study). These results again suggest that the monomer dimensions are a key factor influencing the water solubility of dental materials. The smaller the monomer molecules, the greater the SL value. It is noteworthy that the solubility studies probably do not provide information about the real content of the sol fraction, but that they only provide information about the sol fraction that is released by the polymer matrix.

Testing the dental restorative materials for mechanical properties is essential from an engineering perspective. The flexural strength of the dental material should be maximized, whereas its elastic modulus and hardness should remain similar to those of the surrounding tissues in order to avoid an inadequate stress-transfer on the loading [46,47].

The flexural modulus of the MEBDI-based homopolymers decreased with an increasing number of oxyethylene groups due to the increasing monomer elasticity. For the same reason, an increase in the flexural strength was observed. The E and σ values, shown by the MEBDI-based homopolymers, were usually higher than those of the dental homopolymers, except for TM’s modulus and HM’s flexural strength. The TM stiffness was greater than those of Bis-GMA and UDMA, whilst being similar to that of TEGDMA. The E value shown by TM can be regarded as high when compared to those determined for other ones known from the literature, urethane-dimethacrylates having the TEGMMA wings. Those monomers and their homopolymers were described in our previous study [18]. HM showed a higher *σ* value than UDMA, but a lower one than Bis-GMA and TEGDMA. High values of E and σ, achieved by the MEBDI-based homopolymers, can be attributed to the semi-aromatic character of monomers and a high degree of conversion in their homopolymers. A comparison of the results for both flexural properties leads to the conclusion that DM achieved the best characteristics, as it was characterized by a higher modulus and mechanical strength than those of all the dental dimethacrylates.

The findings of this study on the copolymers confirm a positive effect of the MEBDI chemical structure on the flexural modulus and flexural strength. All the MEBDI-based copolymers had higher E and σ values than the dental copolymers, except for HM:T, which showed a σ value 5% lower than that of Bis-G:U:T. A more detailed analysis shows that the copolymers composed of HM and DM had a higher modulus and flexural strength than those which did not contain DM. When comparing the flexural properties of copolymers by the reactive diluent type, those composed of TM showed higher E and σ values than those composed of TEGDMA. The explanation for this result, in addition to the MEBDI aromatic character and high DC, can be attributed to the influence of the presence of the urethane groups. The literature provides evidence of a positive effect of hydrogen bondings on mechanical properties, particularly those formed by the urethane group [10,11,20]. A comparative analysis of Bis-G:T and Bis-G:U:T showed that the copolymer containing UDMA had a higher modulus and mechanical strength than those which did not have UDMA. New MEBDI-based copolymers had a similar flexural strength to Bis-G:U:T (typically, no statistical significance was observed). A detailed analysis of the flexural strength showed that HM:T had the lowest flexural strength. This result can be attributed to the influence of the presence of TEGDMA, causing a decrease in the concentration of urethane groups in the polymer network. The HM:TM copolymer showed a 14% higher σ value, which can be attributed to its fully urethane-dimethacrylate composition. With the further DM introduction, slight increases of approximately 2% in the flexural strength were observed. The HM:DM:TM copolymer, containing only urethane-dimethacrylates, was characterized by the highest σ value.

Hardness is known as the most DC-sensitive property [48]. It has also been shown that aromatic rings and urethane bonds have a positive effect on hardness by increasing its value [18]. Theoretically, the hardness of the studied MEBDI-based homopolymers should decrease with an increasing number of oxyethylene groups, i.e., with decreasing concentrations of benzene rings, double bonds and urethane bonds [18]. However, HM showed the lowest hardness (HB), which can be attributed to the lowest DC of this homopolymer. An initial lengthening of the oligooxyethylene chain resulted in a hardness increase. As a consequence, DM showed the highest HB, due to the highest DC.

The same trend was observed for copolymers. Bis-G:U:T and the MEBDI-based copolymers had a higher hardness than Bis-G:T. The result for the latter copolymer can be explained by the lack of urethane groups. The analysis of the results also showed that the MEBDI-based copolymers constituted wholly of urethane-dimethacrylates had a greater hardness when compared to those containing TEGDMA. This result can be explained by the lower concentration of urethane groups in the TEGDMA-based copolymers, which causes a decrease in the concentration of hydrogen bonds (physical crosslinks). Finally, copolymers containing both HM and DM monomers showed a lower HB than those not containing DM, due to the greater concentration of oxyethylene units, causing an increase in molecular elasticity. HM:DM:TM had the highest hardness in the examined copolymers. This can be explained by the high DC and high concentrations of aromatic rings, as well as urethane bonds, in the latter copolymer. The lowest hardness for HM:T can be explained by the decreased concentration of urethane-bonds due to the presence of TEGDMA and to a high HM molecular stiffness causing a decrease in the DC.

In summary, it might be said that HM, DM and TM represent an interesting alternative for common dental dimethacrylates. However, their application as stand-alone resins in a potential future dental composite matrix shows certain limitations. For example, HM and DM are characterized by a viscosity that is still too high, HM by a low degree of conversion, whereas TM is characterized by excessive water sorption and high polymerization shrinkage. DM stands out among the studied MEBDI-based monomers due to the excellent structural, physico-chemical and mechanical characteristics of its homopolymer. Its only limitation seems to be a relatively high viscosity.

On the other hand, all studied MEBDI-based monomers are an interesting alternative in terms of their use as comonomers for the manufacturing of dental restorative materials. The combinations of HM, DM, TM and TEGDMA used in this study resulted in copolymers characterized by many advantages over typical dental formulations. Their composition could undergo further modifications to meet particular properties of dental restoration, such as high hardness or low water sorption. Therefore, this survey can be treated as a pilot study.

## 4. Materials and Methods

### 4.1. Materials

Bis-GMA (bisphenol A glycerolate dimethacrylate, Sigma-Aldrich, St. Louis, MO, USA), DEG (diethylene glycol, Acros Organics, Geel, Belgium), DMAEMA (2-(dimethyloamino)ethyl methacrylate, Sigma-Aldrich, St. Louis, MO, USA), HEMA (2-hydroxyethyl methacrylate, Sigma-Aldrich, St. Louis, MO, USA), MEBDI (1,3-bis(1-isocyanato-1-methylethyl)benzene, Sigma-Aldrich, St. Louis, MO, USA), MMA (methyl methacrylate, Acros Organics, Geel, Belgium), TEG (triethylene glycol, Acros Organics, Geel, Belgium), TEGDMA (triethylene glycol dimethacrylate, Sigma-Aldrich, St. Louis, MO, USA), UDMA (mixture of urethane-dimethacrylate resin, Sigma-Aldrich, St. Louis, MO, USA), CQ (camphorquinone, Sigma-Aldrich, St. Louis, MO, USA), DBTDL (dibutyltin dilaurate, Fluka), PTZ (phenothiazine, Sigma-Aldrich, St. Louis, MO, USA), methylene chloride (POCH S.A.), chloroform (Chempur, Piekary Śl., Poland), toluene (Chempur, Piekary Śl., Poland), K_2_CO_3_ (potassium carbonate, Chempur, Piekary Śl., Poland), MgSO_4_ (magnesium sulphate, Chempur, Piekary Śl., Poland), TMS (tetramethylsilane, Sigma-Aldrich, St. Louis, MO, USA) and KBr (potassium bromide, spectroscopic grade, Acros Organics, Geel, Belgium) were used as received.

### 4.2. Monomer Synthesis and Sample Preparation

The HM, DM and TM monomers were obtained from a two-stage process, including the synthesis of the oligoethylene glycol monomethacrylates (OEGMMA) and their reaction with MEBDI (Scheme 2). As 2-hydroxyethyl methacrylate (HEMA) is commercially available, only DEGMMA and TEGMMA were synthesized by the transesterification reaction of methyl methacrylate (MMA) with the diethylene glycol (DEG) and triethylene glycol (TEG), according to a procedure previously described in the literature [22,23]. Subsequently, the MEBDI-based urethane-dimethacrylate monomers were synthesized via an addition reaction of OEGMMA to MEBDI, which was similar to a previously described route in the literature [22,23].

**Figure 2:**
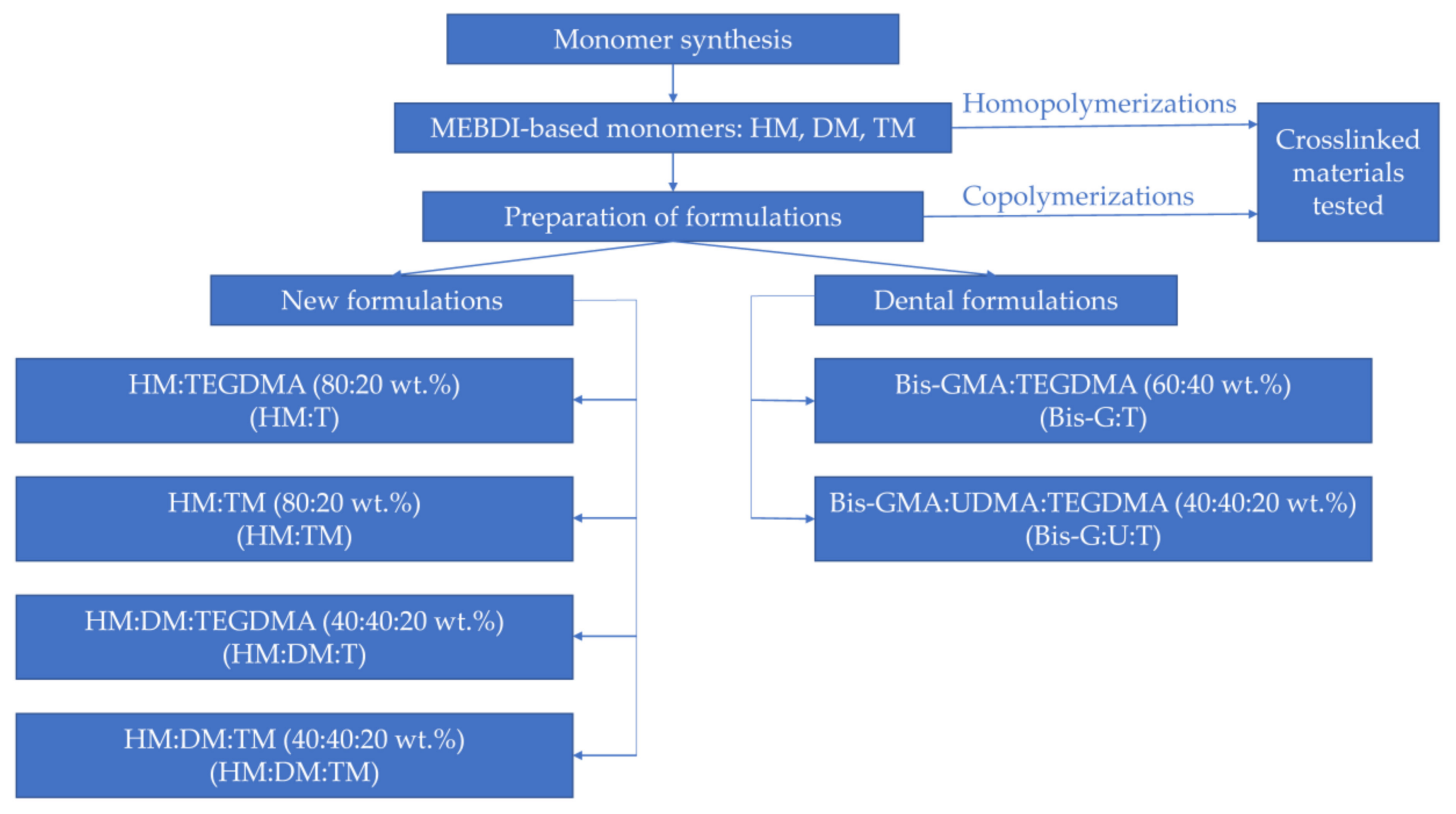
Schematic representation of sample groups tested in this study.

HM, DM and TM were then used to prepare new resin formulations. They were mixed with themselves and TEGDMA in various weight ratios. In total, four mixtures were obtained (Table 1, Figure 2). For comparison purposes, two resin formulations containing typical dental dimethacrylates (Bis-GMA, UDMA and TEGDMA) were prepared.

The monomers and monomer compositions were, respectively, homo- and copolymerized via a photoinitiated process with the use of a CQ/DMAEMA initiating system.

#### 4.2.1. Synthesis of the Oligoethylene Glycol Monomethacrylates

Diethylene glycol monomethacrylate (DEGMMA) and triethylene glycol monomethacrylate (TEGMMA) were synthesized from 1.5 mol of MMA (150.2 g) and 1.0 mol of glycol, according to the procedure described in the literature [22,23]. Respectively, DEG (106.1 g) and TEG (150.2 g) were used for that purpose (Scheme 2). K_2_CO_3_, 8 wt.%, was used as a transesterification reaction catalyst. PTZ, 500 ppm, was used as an inhibitor to prevent the spontaneous radical polymerization of the methyl methacrylate group. The transesterification reaction was carried out in toluene (30 wt.% solution), with the use of a 1000 mL round-bottomed flask equipped with a Vigreux distillation column. The reaction was terminated when the temperature measured at the head of the column reached 110 °C, which was typically achieved after 2.5 h. The reaction mixture was filtered and washed with distilled water in a 2:1 volume ratio. Then, the aqueous layer was extracted with chloroform in a 3:1 volume ratio. The chloroform fraction was dried overnight with MgSO_4_, and then the solvent was removed on a rotary evaporator under reduced pressure (first at 30 mbar and then at 3 mbar). The crude product was distilled under vacuum (2 mbar), taking the boiling fraction of DEGMMA at 100–110 °C and of TEGMMA at 110–120 °C. The final product yields were at 14% and 17%, respectively.

#### 4.2.2. Synthesis of the Urethane-Dimethacrylates

##### The Addition Reaction of HEMA to MEBDI

A 50 wt.% solution of HEMA (26.14 g, 0.20 mol) in methylene chloride, admixed with DBTDL (0.03 wt.%, the catalyst) and PTZ (500 ppm, the inhibitor), was placed in a 250 mL three-neck flask equipped with a mechanical stirrer, thermometer, dropping funnel and condenser. The reaction mixture was heated to 40 °C, and the 50 wt.% solution of MEBDI (24.42 g, 0.10 mol) in methylene chloride was added dropwise for 1 h, maintaining the temperature at 40 °C. Stirring was continued for 3 h at 40 °C. After cooling, the methylene chloride was evaporated under vacuum (first at 30 mbar and then at 3 mbar). HM (Scheme 2) was obtained with a 100% yield. The product was a highly viscous resin with a very slight straw color.

FTIR (KBr): υ = 3361 (s, NH), 3105 (w, =CH_2_), 2977 (s, CH_2_, CH_3_), 1715 (s, C=O), 1637 (m, C=C), 1606 (w, aromatic), 1520 (s, NH), 1455 (s, CH_2_, CH_3_), 1248, 1168 and 1095 (s, C-N, C-O-C), 945 (m, =CH), 778 and 706 (m, =CH aromatic) cm^−1^.

^1^H NMR (300 MHz, CDCl_3_): δ = 1.67 (s, 12H, C(CH_3_)_2_), 1.82 (s, 6H, =C–CH_3_), 4.06–4.20 (m, 8H, CH_2_O), 5.56 (s, 2H, NHCOO), 5.62 and 6.09 (2s, 4H, =CH_2_), 7.22 (m, 3H, aromatic CH), 7.45 (t, 1H, aromatic CH) ppm.

^13^C NMR (300 MHz, CDCl_3_): δ = 18 (=C–CH_3_), 30 (C(CH_3_)_2_), 56 (C(CH_3_)_2_), 62 (NH-COO-CH_2_), 63 (CH_2_-CH_2_-OCO), 122, 124, 128 and 146 (aromatic CH=), 126 (CH_2_=), 136 (=C–CH_3_), 156 (NH-COO), 168 (C=O) ppm.

##### The Addition Reaction of DEGMMA to MEBDI

The DM synthesis was performed in an analogous way to that of HM, starting with 53.18 g (0.31 mol) of DEGMMA and 37.33 g (0.15 mol) of MEBDI. DM (Scheme 2) was obtained with a 100% yield. It was a viscous resin with a very slight straw color.

FTIR (KBr): υ = 3359 (s, NH), 3105 (w, =CH_2_), 2976 (s, CH_2_, CH_3_), 1716 (s, C=O), 1637 (m, C=C), 1605 (w, aromatic), 1524 (s, NH), 1456 (s, CH_2_, CH_3_), 1249, 1170 and 1086 (s, C-N, C-O-C), 947 (m, =CH), 779 and 707 (m, =CH aromatic) cm^−1^.

^1^H NMR (300 MHz, CDCl_3_): δ = 1.65 (s, 12H, C(CH_3_)_2_), 1.95 (s, 6H, =C-CH_3_), 3.61–3.83 (m, 8H, CH_2_O), 4.12 (m, 4H, CH_2_-OCONH), 4.32 (m, 4H, CH_2_-OCO), 5.43 (s, 2H, NHCOO), 5.61 and 6.23 (m, 4H, =CH_2_) 7.25 (m, 3H, aromatic CH=), 7.51(t, 1H, aromatic CH=) ppm.

^13^C NMR (300 MHz, CDCl_3_): δ = 18 (=C-CH_3_), 30 (C(CH_3_)_2_), 55 (C(CH_3_)), 63 (NH-COO-CH_2_), 64 (-CH_2_-CH_2_-COO), 68–69 (CH_2_-O), 122, 124, 128 and 147 (aromatic CH=), 126 (CH_2_=), 136 (=C-CH_3_), 154 (NH-COO), 168 (C=O) ppm.

##### The Addition Reaction of TEGMMA to MEBDI

The TM synthesis was performed in an analogous way to those of HM and DM, starting from 41.67 g (0.19 mol) of TEGMMA and 23.53 g (0.10 mol) of MEBDI. TM (Scheme 2) was obtained with a 100% yield. As previously, it was a viscous resin with a very slight straw color.

FTIR (KBr): υ = 3354 (s, NH), 3105 (w, =CH_2_), 2985–2950 (s, CH_2_, CH_3_), 1716 (s, C=O), 1637 (m, C=C), 1605 (w, aromatic), 1524 (s, NH), 1455 (s, CH_2_, CH_3_), 1249, 1171 and 1085 (s, C-N, C-O-C), 947 (m, =CH), 779 and 707 (m, =CH aromatic) cm^−1^.

^1^H NMR (300 MHz, CDCl_3_): δ = 1.66 (s, 12H, C(CH_3_)_2_), 1.82 (s, 6H, =C-CH_3_), 3.52–3.81 (m, 16H, CH_2_-O), 4.21 (m, 4H, CH_2_-OCONH), 4.36 (m, 4H, CH_2_-OCO), 5.62 (s, 2H, NHCOO), 5.65 and 6.24 (2s, 4H, =CH_2_), 7.21 (m, 3H, aromatic CH=), 7.66(t, 1H, aromatic CH=) ppm.

^13^C NMR (300 MHz, CDCl_3_): δ = 18 (=C-CH_3_), 30 (C(CH_3_)_2_), 55 (C(CH_3_)), 63 (NH-COO-CH_2_), 64 (-CH_2_-CH_2_-COO), 69–70 (CH_2_-O), 122, 124, 128 and 146 (aromatic CH=), 126 (CH_2_=), 136 (=C-CH_3_), 156 (NH-COO), 168 (C=O) ppm.

#### 4.2.3. Photopolymerization

Curing was performed with the use of the CQ/DMAEMA photoinitiating system. CQ, the photoinitiator, was added in a 0.4 wt.% and DMAEMA, the reducing agent, in the amount of 1 wt.%. Samples containing the initiating system were introduced into the molds, covered with the PET film to reduce the oxygen inhibition effect, and irradiated with a UV-VIS lamp (Ultra Vitalux 300, Osram, Munich, Germany) at room temperature for 60 min. Glass molds with a rectangular shape of 90 mm × 90 mm × 4 mm (length × width × thickness) and disc-like shape of 15 mm × 1.5 mm (diameter × thickness) were used in the curing process.

### 4.3. Nuclear Magnetic Resonance (NMR)

^1^H NMR and ^13^C NMR spectra of the monomers were recorded in the CDCl_3_ solution, using TMS as an internal standard. A 300 MHz NMR spectrometer (UNITY/INOVA, Varian, Palo Alto, CA, USA) was employed for these experiments.

### 4.4. Fourier Transform Infrared Spectroscopy (FTIR) and Degree of Conversion

FTIR spectra were recorded utilizing a Spectrum Two (Perkin-Elmer, Waltham, MA, USA) spectrometer. Monomers were tested in the form of thin layers closed between two KBr pellets. The polymers were ground into a fine powder (sieved to a grain size smaller than 25 µm) and tested in the form of KBr pellets. The spectra of the monomers and corresponding polymers were recorded with 128 scans at a resolution of 1 cm^−1^.

The degree of conversion (DC) was calculated utilizing the internal standard method, according to the following equation:

(1) $$DC\;\left(\%\right)=\left(1-\frac{{\left(\frac{A_{C=C}}{A_{Ar}}\right)}_{polymer}}{{\left(\frac{A_{C=C}}{A_{Ar}}\right)}_{monomer}}\right)\times 100$$

where A_C=C_ is the absorption band intensity of the carbon-carbon double bond stretching vibrations, located at 1637cm^−1^, and A_Ar_ is the absorption intensity of the internal standard band, corresponding to the C–C skeletal stretching vibrations in the aromatic ring, located at 1605 cm^−1^.

### 4.5. Viscosity

The viscosity (η, Pa∙s) was measured by employing a rotating spindle viscometer (Visco Star Plus L, Brookfield Fungilab Viscometer, Barcelona, Spain) at 20 °C and 45 °C according to the outline in the ISO 2555 standard [49]. The viscosity was measured using an L3 spindle, which allowed for the recording of viscosity values between a 10% and 9 % torque.

### 4.6. Density and polymerization shrinkage

The monomer densities (d_m_) were determined with a pycnometer according to ISO 1675 [50]. The polymer densities (d_p_) were measured utilizing an analytical balance equipped with a density determination kit using Archimedes’ principle. Water was used as an immersion liquid. The analytical balance, with a 0.01 mg accuracy (XP Balance, Mettler Toledo, Greifensee, Switzerland), was used for these measurements.

The experimental polymerization shrinkage (S) was calculated according to the following equation:

(2) $$S\;\left(\%\right)=\left(1-\frac{d_{m}}{d_{p}}\right)\times 100$$

where, d_m_ is the monomer density, and d_p_ is the polymer density.

The theoretical polymerization shrinkage (S_theor_) was calculated, assuming a 100% conversion of the methacrylate double bonds, from the following equation:

(3) $$S_{theor}\;\left(\%\right)=\left(\frac{2\times 22.5\times d_{m}}{MW}\right)\times 100$$

where MW is the monomer molecular weight. A value of 22.5 cm^3^/mol corresponds to a theoretical decrease in volume per one mole of double bonds [34].

### 4.7. Water Sorption and Solubility

The water sorption (WS) and solubility (SL) were determined according to the ISO 4049 standard [35]. Disc-like samples of 15 mm × 1.5 mm (diameter × thickness) were used in these tests. Samples, before testing, were sanded clean with fine sanding paper until smooth, level surfaces were obtained. After that, they were dried at 100 °C in a conditioning oven until a constant weight was achieved. The analytical balance, with a 0.01 mg accuracy (XP Balance, Mettler Toledo, Greifensee, Switzerland), was used in this analysis. The initial weight of each sample was designated as (*m_0_*). Then, the samples were immersed in distilled water and left for seven days at room temperature. After that, the samples were removed from the water, blotted dry and weighed (*m_1_*). *WS* was calculated using the following equation:

(4) $$WS\;\left(\frac{\mu g}{mm^{3}}\right)=\frac{m_{1}-m_{0}}{V}$$

where *m_1_* is the mass of the swollen sample, *m_0_* is the initial mass of the dried sample, and *V* is the initial volume of the dried sample.

After that, the specimens were dried again to a constant weight (*m_2_*), and *SL* was calculated according to the following equation:

(5) $$SL\;\left(\frac{\mu g}{mm^{3}}\right)=\frac{m_{0}-m_{2}}{V}$$

where *m_2_* is the mass of the dried sample after immersion in water.

### 4.8. Mechanical Properties

#### 4.8.1. Flexural properties

The flexural modulus (E) and flexural strength (σ) were determined using the ISO 178 standard [51], utilizing a universal testing machine (Zwick Z020, Ulm, Germany). Samples of 80 mm × 10 mm × 4 mm (length × width × thickness) were cut out of the previously prepared molds and sanded clean with fine sanding paper until smooth, level surfaces were obtained. E and σ were calculated using the following equations, respectively:

(6) $$E\left(MPa\right)=\frac{P_{1}l^{3}}{4bd^{3}\delta }$$

and

(7) $$\sigma \left(MPa\right)=\frac{3Pl}{2bd^{2}}$$

where, *P_1_* is the load at a selected point of the elastic region of the stress–strain plot, *P* is the maximum load, *l* is the distance between supports, *b* is the sample width, *d* is the sample thickness and *δ* is the deflection of the sample at *P_1_*.

#### 4.8.2. Hardness

The hardness (HB) of polymers was determined according to ISO 2039 – 1 [52], by using the VEB Werkstoffprűfmaschinen apparatus (Leipzig, Germany). Tests were performed on disc-like samples of 90 mm × 4 mm (diameter × thickness). They were cut out from the previously prepared molds and sanded clean until smooth, level surfaces were obtained. *HB* was calculated according to the equation:

(8) $$HB\left(MPa\right)=\frac{F_{m}\frac{0.21}{\left(h-h_{r}\right)+0.21}}{\pi dh_{r}}$$

where *F_m_* is the test load, *d* is the diameter of the ball intender (*d* = 5 mm), *h* is the immersion depth and *h_r_* is the reduced depth of impression *(h_r_ =* 0.25 mm).

### 4.9. Statistical Analysis

The experimental results were analyzed using a one-way analysis of variance (ANOVA). Pair-wise comparisons were conducted through the Student’s t-test with a significance level (p) of 0.05. The TTEST function in Microsoft Excel 2016 was used for that purpose. For each physical property, a set of five samples was tested. The results were expressed as average values, with their associated standard deviations (SD).

## 5. Conclusions

The newly developed MEBDI-based urethane-dimethacrylate formulations achieved promising physico-chemical and mechanical characteristics so as to be suitable to be applied as dental composite matrices. Additionally, new fundamental knowledge of the structure-property relationships in dimethacrylate polymers has been proposed. This can be helpful in the designing process of other new urethane-dimethacrylate monomers for dentistry.

None of the studied MEBDI-based urethane-dimethacrylate monomers could be used in their pure form in a composite matrix. Monomers obtained from 2-hydroxyethyl methacrylate and diethylene glycol monomethacrylate have an excessive viscosity. The monomer obtained from triethylene glycol monomethacrylate resulted in a polymer with an excessive water sorption.

A combination of the MEBDI-based urethane-dimethacrylates resulted in copolymers with a high degree of conversion, low polymerization shrinkage, low water sorption and water solubility, and good mechanical properties. The application of TEGDMA resulted in advantages such as a decrease in the composition viscosity and copolymer water sorption.

## Figures and Tables

**Table 1:** The sample names and compositions. The product of the addition reaction of HEMA to MEBDI is abbreviated to HM, product of the addition reaction of DEGMMA to MEBDI is abbreviated to DM, product of the addition reaction of TEGMMA to MEBDI is abbreviated to TM, UDMA is abbreviated to U, Bis-GMA to Bis-G, and TEGDMA to T.

Sample Name		Composition					
		HM	DM	TM	Bis-G	T	U
HM	wt.%	100.0					
	mol%	100.0					
DM	wt.%		100.0				
	mol%		100.0				
TM	wt.%			100.0			
	mol%			100.0			
U	wt.%						100.0
	mol%						100.0
Bis-G:T	wt.%				60.0	40.0	
	mol%				45.7	54.3	
Bis-G:U:T	wt.%				40.0	20.0	40.0
	mol%				33.5	30.0	36.5
HM:T	wt.%	80.0				20.0	
	mol%	69.4				30.6	
HM:TM	wt.%	80.0		20.0			
	mol%	84.6		15.4			
HM:DM:T	wt.%	40.0	40.0			20.0	
	mol%	36.6	31.0			32.4	
HM:DM:TM	wt.%	40.0	40.0	20.0			
	mol%	45.1	38.3	16.6			

**Table 2:** Properties of the studied monomers and their mixtures: molecular weight (MW), the concentration of double bonds (X_DB_), density (d_m_) and viscosity (η) – measured at 20 °C and 45 °C. All density results were statistically insignificant (*p* > 0.05), except for one couple which revealed a statistically significant result (*p* ≤ 0.05) and this is marked with the letter *a*. The results for the viscosity measurements were statistically significant (*p* ≤ 0.05). Couples that revealed statistically insignificant results (*p* > 0.05) for viscosity are highlighted with the letters a’–d’.

Monomer	MW (g/mol)	X_DB_ (mol/kg)	d_m_ (g/cm^3^)		η (20 ⁰C) (Pa∙s)		η (45 ⁰C) (Pa∙s)	
			Average	SD	Average	SD	Average	SD
HM	504.6	3.96	1.15	0.08	- ^2^	-	18.75	0.96
DM	592.8	3.37	1.15	0.06	19.84	0.47	2.89	0.16
TM	681.2	2.94	1.12	0.07	2.58	0.03	0.52	0.11
Bis-G	512.6	3.90	1.15 ^1^	-	1200 ^3^	-	-	-
U	470.6	4.25	1.11	0.03 ^a^	8.44	0.11	1.38	0.03 ^a’,b’^
T	286.3	6.99	1.09 ^1^	-	0.01 ^3^	-	-	-
Bis-G:T	408.5	4.90	1.14	0.07	0.54	0.03	0.17	0.12
Bis-G:U:T	429.5	4.66	1.13	0.04	5.91	0.08	1.77	0.12
HM:T	438.0	4.57	1.13	0.06	4.03	0.08	1.21	0.17 ^a’,c’^
HM:TM	531.8	3.76	1.14	0.04	7.25	0.06	1.46	0.14 ^b’^
HM:DM:T	461.1	4.34	1.14	0.03 ^a^	2.82	0.05	0.85	0.06 ^d’^
HM:DM:TM	568.1	3.52	1.14	0.04	5.06	0.06	1.02	0.15 ^c’,d’^

^1^ As cited in Ref [18]. ^2^ Unable to be measured. ^3^ As cited in Ref [19].

**Table 3:** The degree of conversion (DC) and polymerization shrinkages, experimentally measured (S) and theoretically calculated (S_theo*r*_) in the studied polymers. The majority of the results were statistically significant (*p* ≤ 0.05). Couples, that revealed statistically insignificant results (*p* > 0.05) were highlighted with letters: a–m – for the DC and a’–j’ – for the S.

Polymer	DC (%)		S (%)		S_theor_ (%)	
	Average	SD	Average	SD	Average	SD
HM	57.7	5.6 ^a^	3.03	0.10 ^a’^	10.25	0.07
DM	89.0	3.9 ^b^	4.31	0.13 ^b’,c’^	8.72	0.11
TM	88.4	5.1 ^b^	4.12	0.14 ^d’,e’,f’^	7.83	0.06
Bis-G	34.5 ^1^	6.0	4.71 ^2^	0.58	10.11 ^1^	0.06
U	77.6	2.9 ^c,d,e^	2.98	0.36 ^a’^	10.65	0.17
T	82.5 ^1^	4,0	11.16 ^2^	0.08	17.12 ^1^	0.02
Bis-G:T	61.2	4.7 ^a,f,g^	6.06	0.23 ^g’^	12.53	0.06
Bis-G:U:T	69.3	5.2 ^f,h,i,j,k^	4.23	0.12 ^d’,h’,i’^	11.79	0.18
HM:T	73.6	6.5 ^c,h,l^	4.06	0.16 ^b’,e’,h’,j’^	11.62	0.13
HM:TM	65.2	4.9 ^g,i,m^	4.14	0.35 ^c’,f’,i’,j’^	9.67	0.16
HM:DM:T	72.1	5.4 ^d,j,l,m^	3.74	0.16	11.12	0.27
HM:DM:TM	80.7	6.1 ^e,k^	6.31	0.23 ^g’^	9.06	0.12

^1^ As cited in Ref [33]. ^2^ As cited in Ref [18].

**Table 4:** The water sorption (WS) and water solubility (SL) in the studied polymers. The majority of the results are statistically significant (*p* ≤ 0.05). Couples that have statistically insignificant results (*p* > 0.05) are highlighted with the letters: a–g – for the WS and a’–g’ – for the SL.

Polymer	WS (µg/mm^3^)		SL (µg/mm^3^)	
	Average	SD	Average	SD
HM	13.34	2.01 ^a^	3.13	0.17 ^a’^
DM	21.45	1.27 ^b,c^	6.82	0.26 ^b’^
TM	46.01	2.06	6.31	0.21 ^c’,d’,e’^
Bis-G	33.49 ^1^	-	10.44 ^1^	-
U	11.34	1.85 ^a^	5.06	0.33 ^f’^
T	69.51 ^1^	-	2.41 ^1^	-
Bis-G:T	26.74	1.22 ^d,e^	3.83	0.43
Bis-G:U:T	25.64	1.20 ^d,f^	5.75	0.33 ^c’^
HM:T	19.34	2.43 ^b,g^	6.49	0.33 ^b’,d’,g’^
HM:TM	32.67	1.26	4.75	0.41 ^f’^
HM:DM:T	20.78	1.32 ^c,g^	6.49	0.21 ^e’,g’^
HM:DM:TM	28.57	2.46 ^e,f^	3.32	0.20 ^a’^

^1^ As cited in Ref [13].

**Table 5:** The mechanical properties of the studied polymers: flexural modulus (E), flexural strength (σ) and hardness (HB). The majority of the results are statistically significant (*p* ≤ 0.05). Pairs which have statistically insignificant results (*p* > 0.05) are highlighted with the letters: a–g – for the *E*, a’–i’ – for the σ, and a’’–k’’ – for the HB.

Polymer	E (MPa)		σ (MPa)		HB (MPa)	
	Average	SD	Average	SD	Average	SD
HM	4657	77	87.4	5.2 ^a’^	151.5	5.7 ^a’’,b’’,c’’^
DM	4454	71^a^	147.0	5.5	179.4	10.0 ^d’’,e’’^
TM	3885	73 ^b^	164.4	5.2	172.2	8.6 ^f’’,g’’,h’’^
Bis-G	3872 ^1^	215	110.0^1^	7.0	73.1^1^	4.0
U	3352	104 ^c^	79.3	4.3 ^b’^	173.8	4.6 ^d’’,f’’^
T	3910 ^1^	199	89.0 ^1^	6,0	129.3^1^	7.9
Bis-G:T	3328	74 ^c^	78.0	5.3 ^b’^	96.6	4.8
Bis-G:U:T	3849	87 ^b^	102.7	11.1 ^a’,c’,d’,e’,f’^	159.7	11.7 ^a’’,g’’,i’’,j’’^
HM:T	4142	124 ^d,e^	97.8	5.7 ^c’^	122.1	13.8
HM:TM	4194	124 ^d,f^	111.2	6.2 ^d’,g’,h’^	152.1	12.9 ^b’’,h’’,i’’,k’’^
HM:DM:T	4241	71 ^e,f,g^	112.9	10.9 ^e’,g’,i’^	144.5	9.0 ^c’’,j’’,k’’^
HM:DM:TM	4395	149 ^a,g^	115.4	9.7 ^f’,h’,i’^	183.2	7.6 ^e’’^

^1^ As cited in Ref [18].

## Abbreviations

Bis-G	bisphenol A glycerolate dimethacrylate
Bis-GMA	bisphenol A glycerolate dimethacrylate
Bis-EMA	bisphenol A ethoxylate dimethacrylate
Bis-G:T	copolymer obtained by photopolymerization of Bis-GMA and TEGDMA in the 60:40 weight ratio
Bis-G:U:T	copolymer obtained by photopolymerization of Bis-GMA, UDMA and TEGDMA in the 40:20:40 weight ratio
BPA	bisphenol A
BPO	benzoyl peroxide
CHMDI	4,4′-diisocyanatodicyclohexylmethane
CQ	camphorquinone
DBTDL	dibutylin dilaurate
DC	degree of conversion
DEG	diethylene glycol
DEGMMA	diethylene glycol monomethacrylate
d_m_	monomer density
DM	monomer obtained by the addition reaction of DEGMMA to MEBDI
DMAEMA	N,N-dimethylaminoethyl methacrylate
E	flexural modulus
FTIR	Fourier Transform Infrared Spectroscopy
IPDI	isophorne diisocyanate
HB	hardness
HEMA	2-hydroxyethyl methacrylate
HM	monomer obtained by the addition reaction of HEMA to MEBDI
HMDI	1,6-isocyanatohexane
HM:DM:T	copolymer obtained by photopolymerization of HM, DM and TEGDMA in the 40:40:20 weight ratio
HM:DM:TM	copolymer obtained by photopolymerization of HM,DM and TM in the 40:40:20 weight ratio
HM:T	copolymer obtained by photopolymerization of HM and TEGDMA in the 80:20 weight ratio
HM:TM	copolymer obtained by photopolymerization of HM and TM in the 80:20 weight ratio
MEBDI	1,3-bis(1-isocyanato-1-methylethyl)benzene
MDI	bis-(4-isocyanatophenyl)methane
MMA	methyl methacrylate
MW	molecular weight
OEGMMA	oligoethylene glycol monomethacrylates
PET	polyethylene terephthalate
PTZ	phenothiazine
S	polymerization shrinkage (experimentally determined)
SL	water solubility
S_theor_	polymerization shrinkage (theoretically calculated)
T	triethylene glycol dimethacrylate
TDI	2,4-diisocyanatotoluene
TEG	triethylene glycol
TEGDMA	triethylene glycol dimethacrylate
TEGMMA	triethylene glycol monomethacrylate
TM	monomer obtained by the addition reaction of TEGMMA to MEBDI
TMDI	2,2,4-trimethyl-1,6-diisocyanatohexane
TMS	Tetramethylsilane
TTEGMMA	tetraethylene glycol monomethacrylate
U	1,6-bis-(methacryloyloxy-2-ethoxycarbonylamino)-2,4,4′-trimethylhexane
UDMA	1,6-bis-(methacryloyloxy-2-ethoxycarbonylamino)-2,4,4′-trimethylhexane
WS	water sorption
X_DB_	concentration of double bonds
η	Viscosity
σ	flexural strength
